# The skin and soft tissue infections in hematological patients

**DOI:** 10.1097/QCO.0000000000000632

**Published:** 2023-11-03

**Authors:** Riccardo Ungaro, Malgorzata Mikulska

**Affiliations:** a Division of Infectious Diseases, Department of Health Sciences DISSAL, University of Genoa; b Division of Infectious Diseases, IRCCS Ospedale Policlinico San Martino, Genoa, Italy

**Keywords:** central venous catheter, hematological malignancy, hematopoietic stem cell transplant, nocardia, pseudomonas aeruginosa, rapid growing nontuberculous mycobacteria

## Abstract

Skin and soft tissue infections (SSTIs) in patients with hematological malignancies are frequent, but dedicated epidemiological studies are limited. The aim of this review is to provide updated description of the main etiological agents, differential diagnosis, and treatment.

**Recent findings:**

In addition to common causes of bacterial skin infections in any kind of patients, such as streptococci and staphylococci (the letter frequently resistant to methicillin), *Pseudomonas aeruginosa* is a frequent agent in patients with hematological malignancies, with high virulence and typical infection presenting as ecthyma gangrenosum. Among fungi, fusariosis is the mold infection most frequently associated with skin lesions, although other molds and yeasts (including *Candida tropicalis*) should be also considered. External infections associated with central venous catheters are frequent in the hematological setting, and in addition to staphylococci, Gram-negative bacteria, fungi, and even rapid growing nontuberculous mycobacteria should be considered. Immunodeficiency might either blunt the typical inflammatory response and make sign or symptoms less evident, or predispose the patients to rapid progression of skin infection to subcutaneous tissues or dissemination.

**Summary:**

SSTIs in hematology patients can be caused by various infectious agents resulting in similar clinical presentation. Rapid and accurate diagnosis is fundamental in order to reduce morbidity and mortality.

## INTRODUCTION

Skin and soft tissue infections (SSTIs) are frequent but sometimes underestimated complications in the hematological setting, particularly in recipients of hematopoietic stem cell transplant (HSCT). Unlike for other more severe infectious syndromes such as bloodstream infection or pneumonia, there are few systematic descriptions and epidemiological studies of SSTIs in hematology.

Even tough infectious agents are the most frequent cause of skin lesions, other pathologies of noninfectious origin, such as direct invasion of neoplastic cells, paraneoplastic reactions, neutrophilic dermatoses such as Sweet's syndrome and pyoderma gangrenosum, graft-versus-host disease (GvHD), medications (including both traditional chemotherapy and targeted therapies), and allergic reactions should also be considered in the differential diagnosis and constitute important diagnostic challenge [1].

The aim of this review is to provide updated description of the main SSTIs in patients with hematological disorders and to highlight the peculiarity of SSTIs in this setting. Although a separate paper is dedicated to fungal SSTI, those typical for hematology patients will be briefly discussed here. The treatment of all the causes of SSTI developing in hematology patients is beyond the scope of this review. 

**Box 1:**
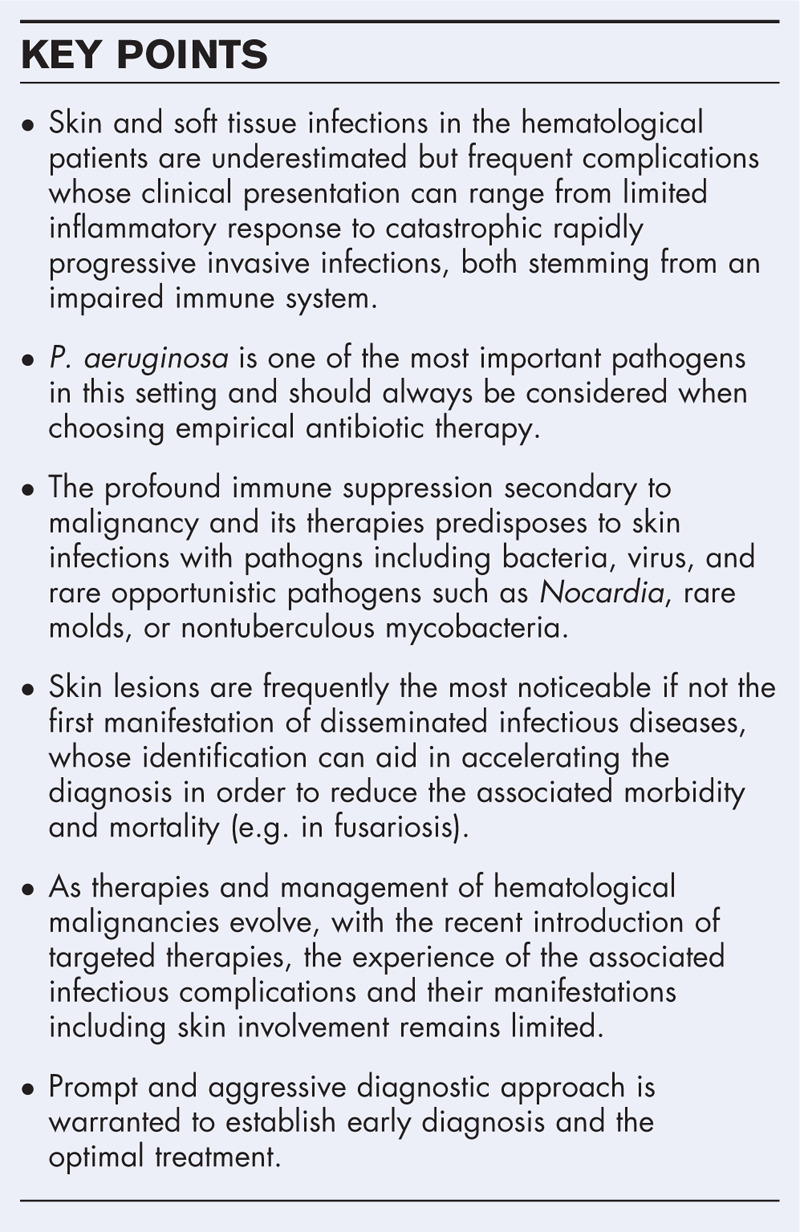
no caption available

## RISK FACTORS AND PARTICULARITIES OF SKIN AND SOFT TISSUE INFECTIONS IN HEMATOLOGY SETTING

Mucositis, epithelial damage and myelosuppression that follow the underlying disease and/or the chemotherapeutic cytotoxic regimen, predispose to bacterial translocation and infections, usually affecting integumental surfaces such as respiratory tract, gastrointestinal tract and skin [2]. Other risk factors for SSTI in this setting are the quasi-universal and prolonged use of central venous catheters, skin microbiome dysbiosis, and poor wound healing because of immunocompromised state. Finally, patients with hematological malignancies, and especially HSCT recipients, might have other comorbidities further predisposing to SSTI such as diabetes (frequently steroid-induced), renal failure, skin fragility caused by GvHD or malnutrition [3,4].

In addition to localized infections such as erysipelas and cellulitis, which are frequently caused by the same pathogens as in immunocompetent individuals, hematologic patients are prone to disseminated fungal, viral and bacterial infections, with metastatic skin localizations that could lead the diagnostic work-up of an unrecognized pathology [5].

Two main issues should be kept in mind when evaluating skin lesions in an immunocompromised patient with a hematological disorder. First, patients frequently have an atypical clinical presentation, ranging from mild signs and symptoms, because of very limited inflammatory response stemming from an impaired immune system [2], to catastrophic rapidly progressive invasive infections with large areas of tissue necrosis involving the fascia caused by the impossibility of immune system to limit the progression of infection [6^▪^,7^▪^]. Second, infrequent causes of skin infection, such as Nocardia, rare molds or nontuberculous mycobacteria (NTM) should always be considered, particularly in patients with a deficit of cellular immunity such as those with HSCT or chronic lymphocytic leukemia (CLL). Therefore, specific dedicated diagnostic methods, frequently involving skin biopsy, are warranted. The main diagnostic procedures indicated in case of SSTIs in patients with hematological disorders are outlined in Table 1.

**Table 1:**
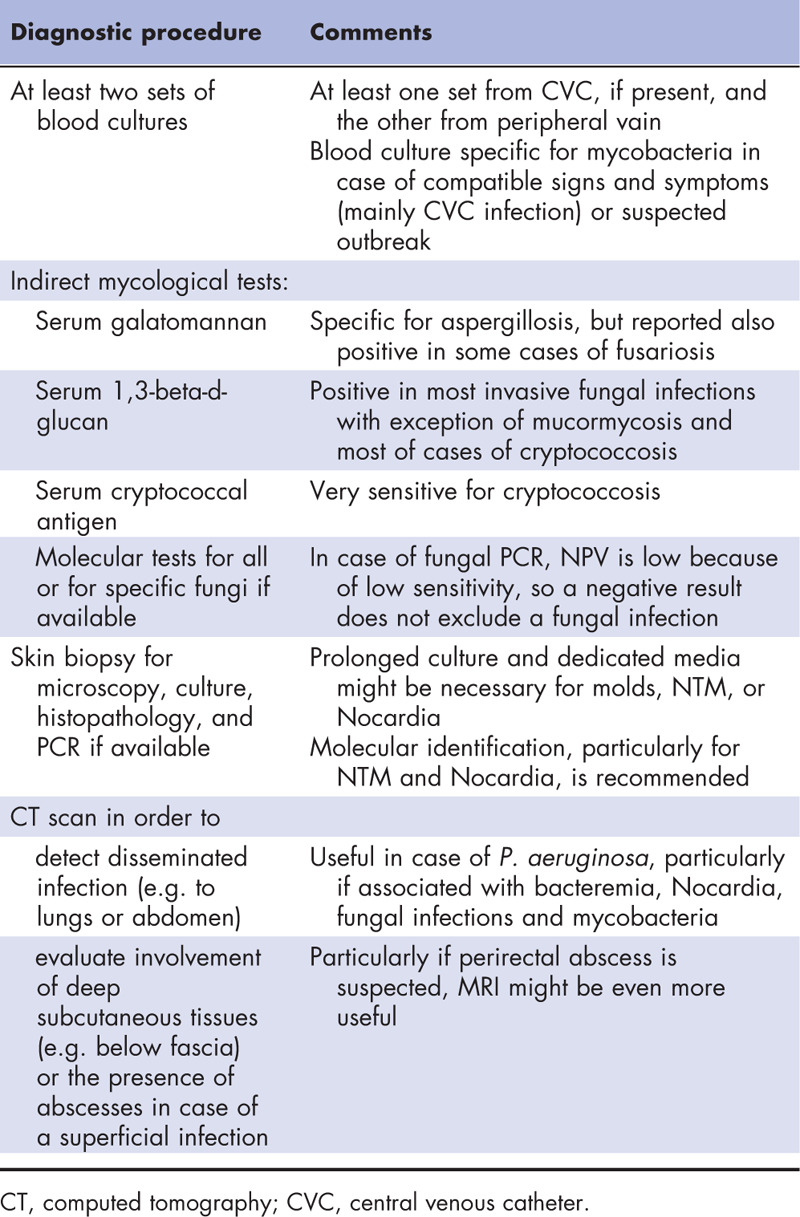
Main diagnostic procedures recommended in case of skin and soft tissues infections in patients with hematological disorders

## BACTERIAL SKIN AND SOFT TISSUE INFECTIONS

Erysipelas, cellulitis, and other soft tissue infections are commonly caused by pyogenic bacteria such as *Streptococcus* spp. and *Staphylococcus aureus*. Cutaneous manifestations, such as areas of localized inflammation with calor, rubor, tumor, and dolor, are usually accompanied by systemic symptoms such as fever, asthenia, and malaise [5]. Localized sings of inflammation might be missing or attenuated, particularly during neutropenia, but may increase significantly upon neutrophil recovery. Because patients with hematological malignancies are rapidly colonized with nosocomial flora and might undergo important microbiota changes due to previous chemotherapies and antibiotic treatments, resistant bacterial strains should be always taken into account when choosing empirical antibiotic therapy.

Immunocompromised patients are at risk of disease progression with dissemination and possible involvement of the subcutaneous structures such as the skeletal muscle or the fascia [8].

### Deep subcutaneous infections

Pyomyositis is an acute intramuscular infection at risk for rapid progression with systemic toxicity, usually caused by methicillin-resistant *S. aureus* (MRSA) and less commonly by streptococci, including *Streptococcus pneumoniae*, presenting with painful swelling and muscular functional impairment. It has been recently described in the context of CLL treated with imatinib and allogenic HSCT [9,10]. In a comprehensive review of 44 cases of pyomyositis associated with hematological malignancy, death occurred in 11.4% of patients [11].

Necrotizing fasciitis is a rare fulminant infection with a progressive destruction of the skin and subcutaneous structure, which can be polymicrobial (anaerobes and coliform bacteria) or monomicrobial (*Streptococcus pyogenes* or *Clostridia* sp.). Atypical bacteria and nosocomial drug-resistant pathogen, such as *Stenotrophomonas maltophilia* or *Acinetobacter* spp., can occur and further complicate the management of this life-threatening infection [12^▪^,^13^]. Immune deficiency related to the malignancy and the concurrent therapies, in particular systemic corticosteroids, rituximab, bortezomib, cyclophosphamide, have been reported to predispose to this syndrome [7^▪^]. Notably, necrotizing polymicrobial fasciitis of the perineum, also known as Fournier's gangrene, could lead to the diagnosis of acute myelogenous leukemia (AML) in patients without obvious predisposing conditions [7^▪^].

### Perianal infections

Perineal abscess is a severe soft tissue infection affecting immunocompromised patients, with an incidence rate up to 10% in leukemic patients with febrile neutropenia [14^▪▪^]. Allogeneic HSCT recipients during the preengraftment period are susceptible to this complication, particularly those with a previous history of anorectal infections [14^▪▪^]. Common clinical symptoms and signs include persistent fever, erythema, and swelling with or without fluctuation and fistula in the perianal region. As with other bacterial infections, local signs may be blunted in neutropenic patients, who usually present only with fever and perirectal pain [15]. Coliform and enteric pathogens are the most frequent organisms recovered from blood cultures or local drainage, followed by *P. aeruginosa* and *Enterococcus* spp. Mortality rate, even with antibiotic treatment and surgical debridement, can reach 2.4% [14^▪▪^,16^▪^].

### Ecthyma gangrenosum

**FIGURE 1:**
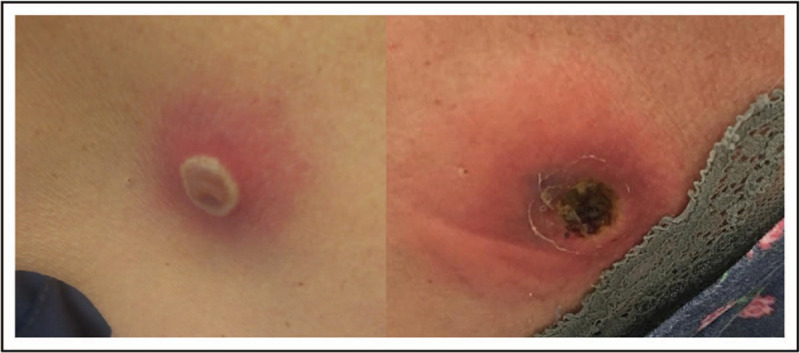
Ecthyma gangrenosum caused by *Pseudomonas aeruginosa* is a neutropenic recipient of allogeneic HSCT, on the second day of infection (left) and 7 days later (right).

*Pseudomonas aeruginosa* is an important pathogen in neutropenic patients, causing 5–10% of bloodstream infections [17]. Ecthyma gangrenosum is a form of necrotizing vasculitis usually caused by *P. aeruginosa*. This peculiar syndrome, typical of the hematological setting, is associated with high mortality and early recognition is crucial for guiding antibiotic treatment. The skin lesion(s) commonly presents as an erythema surrounded-nodule eventually evolving to a necrotic ulcer with a central black eschar (Fig. 1). A systematic review of 167 cases of ecthyma gangrenosum, described in literature from 1975 to 2014, reported *P. aeruginosa* as the primary pathogen isolated either in blood or skin specimen cultures (74% of cases, 59% with concurrent bloodstream infection); nevertheless other bacterial causes were detected in 17% of cases and fungi in 9% [18]. In particular, *Escherichia coli*, *Klebsiella pneumoniae*, *Streptococcus* spp. and *Fusarium* have been reported as possible causative agents other than *P. aeruginosa*[19,20^▪^,21^▪^]. Ecthyma gangrenosum can appear anywhere in the body but most commonly affects the anogenital and axillary areas, followed by the extremities, trunk and face. In individuals with the bacteremic form of ecthyma gangrenosum, the source of infection and other localization (CVC or pneumonia) must be carefully looked for. Targeted antibiotic therapy may be sufficient, although surgical excision of necrotic lesions or abscesses might be necessary [22^▪^].

### Nocardia

Nocardia, an environmental Gram-positive pathogen, causes a rare but severe infection in immunocompromised patients. Most cases of nocardiosis in the hematologic setting occur after allogeneic HSCT with an incidence rate between 0.3% and 1.7% and a mortality rate that could exceed 50% in disseminated disease [23,24]. Notably, low dose of trimethoprim/sulphametoxazole used for pneumocystosis and toxoplasmosis prophylaxis seems to be inadequate in preventing posttransplant nocardiosis [23,25^▪^]. During disseminated disease, metastatization from lungs to skin and other organs such as brain is frequent. In patients with T-cell suppressed function such as those who underwent HSCT, solid organ transplantation, or high-dose corticosteroid therapy, secondary skin lesions during disseminated disease have been described to occur with a rate between 19% [25^▪^] and 32% [23,26^▪▪^]. Secondary lesions can present as abscesses, nodules with or without ulceration or cellulitis as opposed to primary cutaneous nocardiosis that usually occurs in immunocompetent hosts in the form of mycetoma or lymphocutaneous infection [27,28^▪^], and the trunk and proximal extremities are usually involved. In a recent cohort of 67 immunocompromised patients (21 hematological) with disseminated nocardiosis, 13 had cutaneous metastases, 11 of which were nodules or subcutaneous abscesses [25^▪^].

Isolation of *Nocardia* in culture can be difficult, thus the microbiology laboratory should be informed to provide longer incubation period (up to 2–3 weeks) if nocardiosis is suspected [23]. The recent introduction of 16S PCR-based assay has improved the certainty of diagnosis and allows precise species identification which is fundamental for targeted treatment [23,29^▪^].

### Nontuberculous mycobacteria

Nontuberculous mycobacteria (NTM) infections are increasingly detected in immunocompromised patients, especially in those with a cell-mediated immune deficiency. Availability of improved molecular diagnostic methods, widespread use of immunosuppressive therapies, including HSCT, and improved survival of patients with hematological malignancies have all led to an increase in the incidence of NTM [30,31,32^▪^]. Recently, reports of NTM infections in HSCT recipients and patients with CLL undergoing second line therapies, for example with ibrutinib, have been published [33,34^▪^,35^▪^]. In particular, the incidence of NTM infections is 50–600 times greater in HSCT recipients compared to general population, with allogenic transplantation, high-intensity myeloablative conditioning regimens, and GVHD as main risk factors [35^▪^]. Both rapid and slow-growing NTM have been reported to cause infections in hematologic patients, with *M. avium complex*, *M. chelonae*, *M. haemophilum*, and *M. marinum* as commonly isolated pathogens [30,33,34^▪^,36^▪^,^37^]. The incidence of NTM infections and the species involved are subject to geographical variability [38^▪▪^,^39^].

Skin is commonly involved during NTM disease, either after direct environmental inoculation of the pathogen at the site of trauma or following disseminated infections with hematogenous metastatic spreading to the skin. NTM predilect the skin of distal cooler areas such as joint surfaces and extremities [37]. The lesions appear usually as multiple erythematous violaceous nodules, usually painful, with a subacute progression to necrosis and suppuration, with local lymphadenopathy. Skin biopsy is mandatory for a correct diagnosis, with histopathology showing suppurative inflammation with limited granuloma formation and acid fast bacilli [30].

Infections of CVC with rapidly growing NTM, including outbreaks in hematology patients caused by water contamination, have been reported [40,41].

The management of NTM infections has already been reviewed elsewhere [42,43^▪^] and is beyond the scope of this article but prolonged culture might be needed and molecular methods are required for correct species identification [44^▪^]. Antimicrobial combination therapy is recommended and should follow international guidelines for NTM [43^▪^,44^▪^]. Moreover, debridement and surgical drainage may be needed in case of severe localized skin infections, and CVC removal in case of infection is mandatory [33,36^▪^,44^▪^].

## VIRUSES

Reactivation of latent infections is the most common cause of viral skin infections in hematology, particularly in patients with prolonged T-cell-mediated immunosuppression.

Herpes viruses, such as varicella zoster virus and herpes simplex virus, are the most frequently involved, although the incidence of these infections was significantly reduced with common use of acyclovir or valacyclovir prophylaxis in seropositive patients with acute leukemia, certain chronic lymphoproliferative malignancies such CLL or bortezomib-treated patients with multiple myeloma, and after HSCT [45,46^▪^]. If present, VZV reactivation may often result in disseminated shingles, particularly if not promptly recognized and treated, whereas hemorrhagic lesions may be present in thrombocytopenic patients. In HSCT recipients, particularly during preengraftment phase, maculopapular diffuse rash can be caused by reactivation of HHV-6, and differential diagnosis usually include allergic rash, mainly to medications used, and immune-mediated process, such as engraftment syndrome or GvHD [1,47,48]. Kaposi sarcoma is a rare but well recognized HHV-8-mediated cause of neoplastic skin lesions [49^▪^]. Cutaneous lesions usually present as multiple, pigmented, raised or flat, asymptomatic papules or nodules, which vary in color from pale pink to vivid purple and may evolve into larger plaques. Occasionally, lesions may present as exophytic, painful ulcerated and bleeding nodules. In the most recent literature review of Kaposi sarcoma after HSCT covering the 1987–2018 period, Cesaro *et al.* described an incidence 0.17% in allogeneic and 0.05% in autologous HSCT. The organ most commonly affected was skin (9/13 cases) with purple to violaceus cutaneous nodules, followed by oral mucosa (4/11) and visceral involvement (3/11), with seven patients with more than one organ involvement [50^▪▪^]. Reduction of immunosuppression might be sufficient for complete regression of skin lesions but visceral disease may require chemotherapy and/or radiotherapy [50^▪▪^].

Other viral reactivations leading to skin lesions might be caused by poxvirus (molluscum contagiosum virus) or human papilloma virus (HPV). Usually, molluscum contagiosum presents as single, multiple, or clustered firm rounded papules from 2 to 5 mm, pink or skin-colored, with a shiny and umbilicated surface, occasionally with an erythematous halo [51^▪^]. Recent case reports have described atypical presentations in patients with hematologic malignancies, particularly disseminated lesions surrounded by a white halo sign [52], erythematous-squamous widespread plaques and patches [53^▪^], or multiple nonpruritic papules with hyperkeratosis and scaling [54].

Reactivation of HPV can complicate HSCT procedure. In particular, recipients of allogenic HSCT have an increased risk of cutaneous squamous cell carcinoma development, mainly associated with HPV, with a 20-year posttransplant incidence of 3.4% [55^▪^]. In a recently reported cohort of 82 female HSCT recipients, posttransplant incidence of vulvar/genital condylomas was 7.3% [56^▪^]. Noteworthy, the cumulative proportion of any genital HPV infection increased with time after transplantation (up to 40.9% at 20 years) [56^▪^]. Condylomas, warts, and other HPV-related lesions can affect the cervix, vulva, vagina, penis, anus, oral cavity, pharynx and the skin. Abnormal presentations such as giant cauliflower-like condyloma can arise in the context of severe T-cell dysfunction that follows HSCT [57]. The impact of HPV vaccination in this setting might be very beneficial and well conducted studies are urgently needed [58^▪^].

## FUNGI

Invasive and disseminated fungal infections (IFIs) are typical complications in severely immunocompromised hematology patients. Skin manifestations are frequent in some IFIs and prompt recognition of a possible fungal cause can guide targeted diagnosis and an early treatment [59^▪▪^]. A recent literature review on skin lesions due to fungal infections during neutropenia reported 215 cases published from 1985 to 2018, revealing the following species most commonly associated with cutaneous manifestations: *Fusarium* spp. (30.2%), *Aspergillus* spp. (28.4%), and *Candida* spp. (18.1%), followed by *Rizhopus* or *Mucor* spp. (5.1%) and *Trichosporon* spp (2.3%) [59^▪▪^]. Furthermore, Maddy *et al.*[59^▪▪^] found differences in morphology of skin lesions in case of different fungi, with yeasts such as *Candida* and *Trichosporon* commonly manifesting as diffuse erythematous papules without central necrosis or eschar development, whereas molds presenting with tender nodules that subsequently developed eschar and necrosis.

Among IFIs affecting hematological patients, fusariosis most frequently presents with skin lesions, which are reported in up to 75% of patients with disseminated infection [60^▪▪^,61^▪^]. Typically, multiple painful purple lesions with black necrotic center develop on the extremities, with the differential diagnosis including *P. aeruginosa* ecthyma gangrenosum, and are frequently the only early manifestation of fusariosis. Blood cultures are usually positive, and skin biopsy can further support the diagnosis [61^▪^,62^▪^]. The outcome of this rare but serious infection is usually poor, with a survival rate of 43% at 90-days described in a review of 233 cases [63], probably because of the presence of disseminated disease and a high fungal burden when the first clinical manifestations appear [60^▪▪^]. Nevertheless, the increased use of voriconazole and of combination therapies has led to a 21% increase in survival rate in the last decade [63].

Despite being the most frequent mold causing IFIs in hematology, *Aspergillus* spp. is an uncommon cause of skin lesions which occur in 1–5% of invasive infections [60^▪▪^,^64^]. The most recent literature review that focused on cutaneous manifestations of invasive aspergillosis found metastatic skin lesions being erythematous and disseminated papules or nodules with a scarce propensity to secondary necrosis, in contrast to other molds such as *Mucorales*[64].

Finally, in case of mucormycosis, skin infection is the third most common presentation after rhinocerebral and pulmonary syndromes [65^▪^]. It is usually seen in patients with hematologic malignancies, in particular AML, and has been recently described in a patient undergoing ibrutinib therapy for CLL [66^▪^]. The onset of skin infection is acute, usually during the course of a rhinocerebral or disseminated infection. The infection is usually localized, featuring a necrotic eschar surrounded by erythema involving palate or nasal mucosa and/or zygomatic or orbital region [67]. Disseminated skin lesions are less common, they may involving the extremities, the trunk and possibly the head, and are burdened with a very high mortality rate (50–94%) [65^▪^].

Among invasive yeast infections, cutaneous lesions have been reported for *Candida* and *Trichosporon* and they manifest as diffuse erythematous papules or nodules which usually do not evolve into central necrosis [59^▪▪^,68^▪^]. A recent comprehensive literature review found 100 cases of *Candida* skin lesions occurring in neutropenic patients with disseminated candidiasis and reported that they occur more commonly in the setting of induction therapy for de novo or relapsed acute leukemia (86% of cases) and were mainly maculopapular erythematous lesions disseminated through the trunk and extremities [69^▪▪^]. The species most commonly implicated were *C. tropicalis* (68%) and *C*. *krusei* (15%), followed by *C. albicans* (10%). Particularly, disseminated maculopapular lesions during *C. tropicalis* infection occurred in 40% of neutropenic patients without fluconazole prophylaxis, while papules and nodules were less common during *C. krusei* disseminated infection in patients receiving fluconazole prophylaxis. Mortality rate associated with these manifestations during candidemia was high (45.4%) [69^▪▪^]. Among the included studies, only five mainly old studies reported the incidence of skin lesions during candidemia in neutropenia and it was in median 35.8%, ranging from 11.5 to 44% [69^▪▪^].

Finally, *Cryptococcus neoformans* is an uncommon pathogen in the hematologic patients, possibly because of widespread use of fluconazole prophylaxis, affecting those with severe numeral or functional lymphopenia such as in course of CLL. Skin manifestations are not common, compared to lung or meningeal involvement, with multiple skin-colored to pink round papules with an erythematous rim similar to molluscum contagiosum [70^▪^].

## CENTRAL VENOUS CATHETER

The use of central venous catheters (CVCs) can be complicated by local cutaneous infections with or without concomitant bloodstream infections [71^▪^,^72^]. CVCs infection rates varies from 0.018 events/1000 catheter days to 0.35 events/1000 catheter days in adult cancer patients, with higher percentages in the population with hematologic malignancies [71^▪^]. According to guideline definitions, local infections of CVCs include localized entrance or exit-site infections (with erythema and local discharge), infections of the tunneled tract (with tenderness, induration, and erythema along the subcutaneous tract of the catheter for more than 2 cm) and/or infections of the pocket (with tenderness, induration, and erythema of the pocket; purulent drainage with possible device rupture; erosion of overlying skin) [71^▪^,^73^]. Pathogens most commonly responsible of CVCs-related infections (recovered from blood cultures or local swabs [74]) are coagulase-negative staphylococci, *S. aureus* and *Candida*, however there has been an increase in infections caused by gram-negative bacteria (in particular *P. aeruginosa*, *K. pneumoniae*, *E. coli*, and *Acinetobacter* spp.) and rapid-growing mycobacteria [71^▪^,^72^,75^▪^,76^▪^]. When a localized infection is suspected, cultures of skin swab and discharge or drainage, if present, should be performed; concomitant CVC and peripheral vein blood cultures should be obtained. If a complicated localized infection of the tunnel and/or the pocket is diagnosed, removal of the catheter accompanied by targeted antimicrobial therapy is mandatory [72].

Chlorhexidine bathing has been reported effective against CVC-related infections [77^▪^], reducing the risk of catheter colonization, decreasing the incidence of CVC-related bloodstream infection and of exit-site infections of tunneled intravascular catheters, especially among neutropenic patients [77^▪^,^78^].

## CONCLUSION

SSTIs in hematology patients can be caused by numerous different infectious agents resulting in similar clinical presentation. Immune system alteration predisposes to infrequent opportunistic infections and to atypical clinical presentation of common syndromes, further complicating the scenario. Rapid and accurate diagnosis is fundamental in order to reduce the morbidity and mortality in this setting.

## Acknowledgements

*None.*

### Financial support and sponsorship

*None.*

### Conflicts of interest

*M.M. received speaker and board member fees from Pfizer, MSD, Biotest, Gilead and Janssen, all outside the submitted work. R.U. has no conflicts of interest.*

## REFERENCES AND RECOMMENDED READING

Papers of particular interest, published within the annual period of review, have been highlighted as:

▪ of special interest
▪▪ of outstanding interest
